# Modified Albert–Lembert coloanal anastomosis with diverting ileostomy versus Turnbull–Cutait pull-through delayed coloanal anastomosis after intersphincteric resection for low rectal cancer: a single-center retrospective comparative study

**DOI:** 10.1186/s12957-026-04565-3

**Published:** 2026-08-27

**Authors:** Mengzhe Li, Hong Liang, Xi Wang, Zhanpeng Yang, Longshuai Yang, Gaojie Lian, Jianhui Li, Qingwen Fan, Chao Zhang

**Affiliations:** 1 People’s Hospital of Zhengzhou University, Henan Provincial People’s Hospital, Zhengzhou, Henan 450003 China; 2 Henan Provincial People’s Hospital, Zhengzhou, Henan China; 3 Henan University People’s Hospital, Henan Provincial People’s Hospital, Zhengzhou, Henan China

**Keywords:** Modified Albert–Lembert coloanal anastomosis, Turnbull–Cutait pull-through delayed coloanal anastomosis, Intersphincteric resection, Low rectal cancer

## Abstract

**Background:**

Reconstruction after intersphincteric resection (ISR) for low rectal cancer remains technically challenging. Turnbull–Cutait pull-through delayed coloanal anastomosis (DCAA) has been used as a sphincter-preserving delayed reconstructive strategy, but it requires exteriorization of the colon and a planned second-stage perineal procedure. To improve anastomotic stability after ISR, we performed modified Albert–Lembert coloanal anastomosis (MALA) with diverting ileostomy. This study aimed to compare the short-term clinical outcomes of MALA with diverting ileostomy versus DCAA after ISR for low rectal cancer.

**Methods:**

This single-center retrospective comparative study was conducted at Henan Provincial People’s Hospital. Between February 2023 and October 2025, consecutive patients with low rectal cancer who underwent laparoscopic ISR followed by hand-sewn coloanal anastomosis were reviewed. Among them, 58 patients underwent Turnbull–Cutait pull-through delayed coloanal anastomosis (DCAA group), and 42 patients underwent modified Albert–Lembert one-stage coloanal anastomosis with diverting ileostomy (MALA group). Clinical outcomes were compared between the two groups. The primary outcome was overall postoperative morbidity within 30 days after the first surgery.

**Results:**

A total of 100 patients were included, with 58 in the DCAA group and 42 in the MALA group. Baseline demographic, clinical, and tumor characteristics were comparable between the two groups. The 30-day overall postoperative morbidity rates were 32.8% and 23.8% in the DCAA and MALA groups, respectively, with no statistically significant between-group difference (*P* = 0.378). No statistically significant differences were observed in the rates of Clavien–Dindo grade I–II complications (19.0% vs. 16.7%, *P* = 0.799) or grade III or higher complications (13.8% vs. 7.1%, *P* = 0.350). Operative time (201.8 ± 28.6 min vs. 206.5 ± 32.4 min, *P* = 0.463), intraoperative blood loss [60 mL (range, 30–150) vs. 60 mL (range, 30–110), *P* = 0.408], harvested lymph nodes [19 (range, 15–23) vs. 22 (range, 16–24), *P* = 0.356], R0 resection rate (100% vs. 100%, *P* = 1.000), and postoperative hospital stay [15 days (range, 13–21) vs. 14 days (range, 12–20), *P* = 0.225] did not differ significantly between the two groups.

**Conclusions:**

MALA with diverting ileostomy is a technically feasible sphincter-preserving reconstructive strategy in selected patients undergoing laparoscopic ISR for low rectal cancer, and no statistically significant difference in 30-day postoperative morbidity was observed between the MALA and DCAA groups.

## Introduction

Colorectal cancer is one of the most common malignancies worldwide. According to GLOBOCAN 2022 data, colorectal cancer ranks third in incidence and second in cancer-related mortality globally, with rectal cancer accounting for approximately 40% of all colorectal cancer cases [1]. Intersphincteric resection (ISR) is an important sphincter-preserving surgical option for selected patients with low rectal cancer. By combining total mesorectal excision with partial, subtotal, or total resection of the internal anal sphincter, ISR can avoid the permanent stoma associated with abdominoperineal resection while maintaining the possibility of oncological radicality and functional sphincter preservation [2].

However, reconstruction after ISR remains technically challenging in terms of both anastomotic safety and functional preservation, particularly in patients with tumors located 2–5 cm from the anal verge [3, 4]. Because the anastomotic level is extremely low, stapled anastomosis may be technically difficult or infeasible. Therefore, hand-sewn coloanal anastomosis remains indispensable for ultra-low coloanal reconstruction after ISR [5].

Turnbull–Cutait pull-through delayed coloanal anastomosis (DCAA) has been reintroduced as a sphincter-preserving delayed reconstructive strategy for low rectal cancer [6, 7]. By exteriorizing the proximal colon through the anal canal and completing the coloanal anastomosis during a planned second-stage perineal procedure, DCAA may avoid routine diverting ileostomy and reduce the clinical consequences of early anastomotic failure [6–8]. Nevertheless, this technique requires management of an exteriorized colonic segment and may be associated with ischemia, retraction, or necrosis of the exteriorized colon [7]. Therefore, alternative reconstructive strategies that improve anastomotic stability while maintaining acceptable short-term safety remain clinically relevant.

In 1826, Lembert proposed and established the principle of serosal apposition in intestinal anastomosis. The vertical mattress suture described by Lembert inverts the bowel wall at the anastomotic site and may reduce the risk of anastomotic leakage [9]. The subsequent combination of the Lembert suture with the Albert suture, which consists of an inner full-thickness continuous layer and an outer seromuscular mattress layer, formed the classic Albert–Lembert suture. This double-layer anastomosis achieves complete closure of the bowel wall, reduces anastomotic tension, and promotes healing through adequate serosal apposition; it has therefore become a representative technique for hand-sewn anastomosis in gastrointestinal surgery [10].

The classic Albert–Lembert technique is a double-layer bowel anastomosis based on full-thickness approximation with additional seromuscular reinforcement. In the present study, we adapted the principles of layered fixation and reinforcement to modify conventional hand-sewn coloanal anastomosis. This reinforced reconstructive approach was termed modified Albert–Lembert coloanal anastomosis (MALA) and was used for immediate hand-sewn reconstruction after laparoscopic intersphincteric resection for low rectal cancer. In this technique, the seromuscular layer of the proximal colon approximately 5 cm from the resection margin is anchored to the levator hiatus, and the colonic stump is approximated to the external anal sphincter using vertical mattress sutures placed at the intersphincteric groove. This approach is designed to reduce anastomotic tension, prevent proximal colonic retraction, and stabilize the neorectal mucosa, while diverting ileostomy protects the anastomosis during early healing. The present study aimed to compare the short-term clinical outcomes of MALA versus DCAA after laparoscopic ISR for low rectal cancer.

## Methods

### Study design and patients

This retrospective observational study was reported in accordance with the Strengthening the Reporting of Observational Studies in Epidemiology (STROBE) guidelines [11]. We reviewed the clinical data of 100 consecutive patients with low rectal cancer who underwent laparoscopic ISR followed by hand-sewn coloanal anastomosis at Henan Provincial People’s Hospital between February 2023 and October 2025. Patients were divided into two groups according to the reconstructive procedure performed: the DCAA group and the MALA group.

**Fig. 1:**
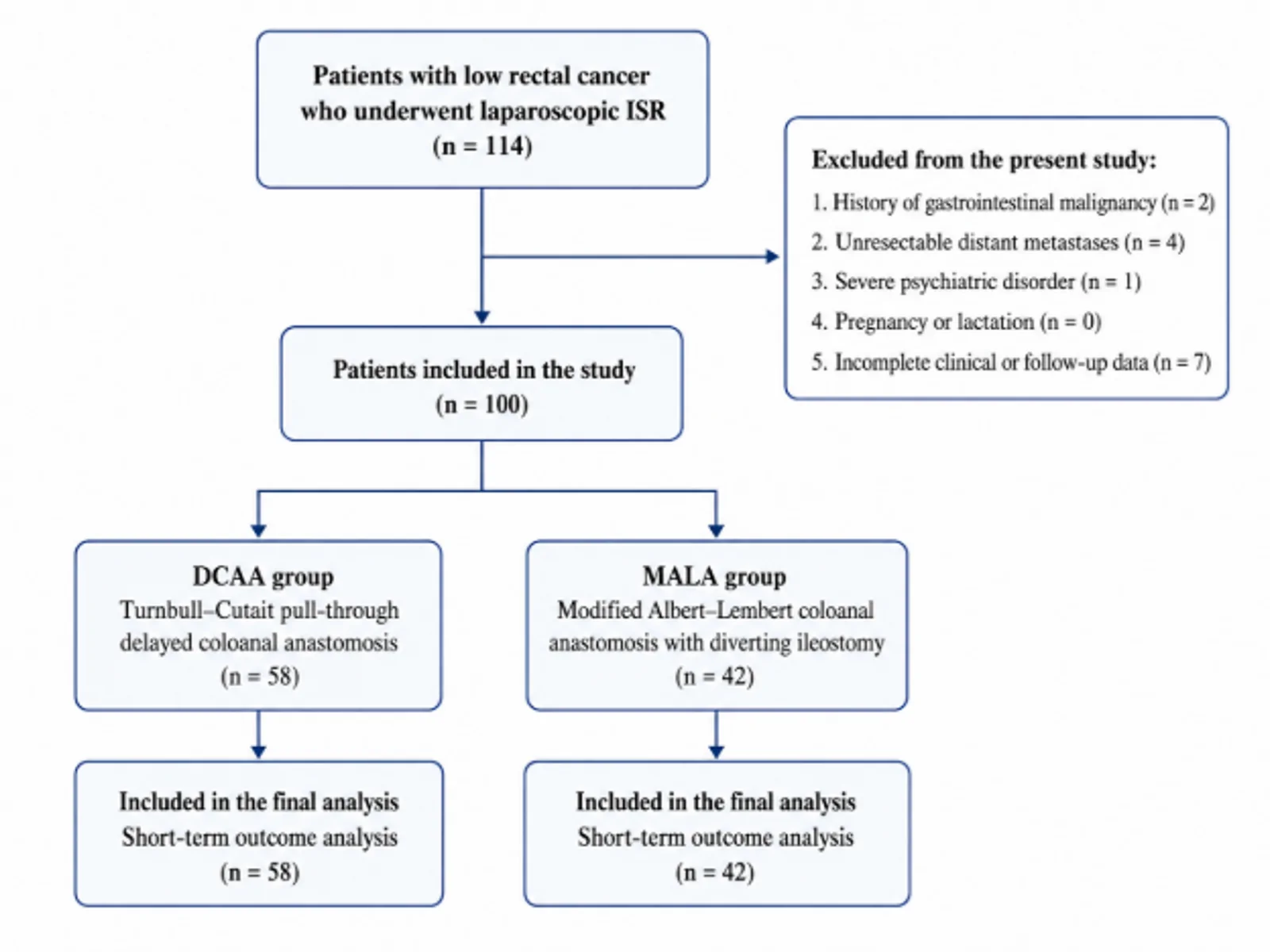
Patient selection flow diagram. ISR, intersphincteric resection; DCAA, Turnbull–Cutait pull-through delayed coloanal anastomosis; MALA, modified Albert–Lembert coloanal anastomosis

The inclusion criteria were as follows: (1) Aged 18–70 years; (2) Tumor located 2–5 cm from the anal verge; (3) Tumor diameter ≤ 5 cm; (4) Well or moderately differentiated adenocarcinoma; (5) Clinical TNM stage: cT1–2N0–2M0; (6) Good preoperative anal continence (Wexner score < 10); (7) Adequate cardiopulmonary function to tolerate laparoscopic surgery; (8) Strong preference for sphincter preservation; (9) No preoperative local radiotherapy; (10) No lateral lymph node enlargement on preoperative imaging; (11) No history of major anorectal diseases. The exclusion criteria were as follows: (1) History of gastrointestinal malignancy; (2) Unresectable distant metastases on preoperative imaging; (3) Severe psychiatric disorder; (4) Pregnancy or lactation; (5) Incomplete clinical or follow-up data. After the potential benefits and risks of both reconstructive options had been explained, the final procedure was selected through shared decision-making between the patient and the surgical team. All operations were performed by the same experienced colorectal surgical team. This study was approved by the Ethics Committee of Henan Provincial People’s Hospital, and written informed consent was obtained from all patients before surgery. The patient selection process is shown in Fig. 1.

### Preoperative assessment

All patients underwent standardized preoperative evaluation, including digital rectal examination, colonoscopy with biopsy, pelvic magnetic resonance imaging, chest and abdominal computed tomography, serum tumor marker measurement, and routine laboratory tests. Clinical staging was determined according to the TNM classification of the 8th edition of AJCC. The indication for ISR was discussed by a multidisciplinary team based on tumor location, sphincter involvement, circumferential resection margin status, baseline anal function, and feasibility of sphincter preservation.

Mechanical bowel preparation (MBP) was performed in the afternoon before surgery, followed by oral non-absorbable antibiotics covering Gram-negative and anaerobic bacteria (such as gentamicin combined with metronidazole) in three divided doses over approximately 10 h. Prophylactic antibiotics with a second-generation cephalosporin (cefuroxime) were intravenously administered within 30 min before surgery [12].

### Surgical procedures

#### MALA group

##### Transabdominal procedure

**Fig. 2:**
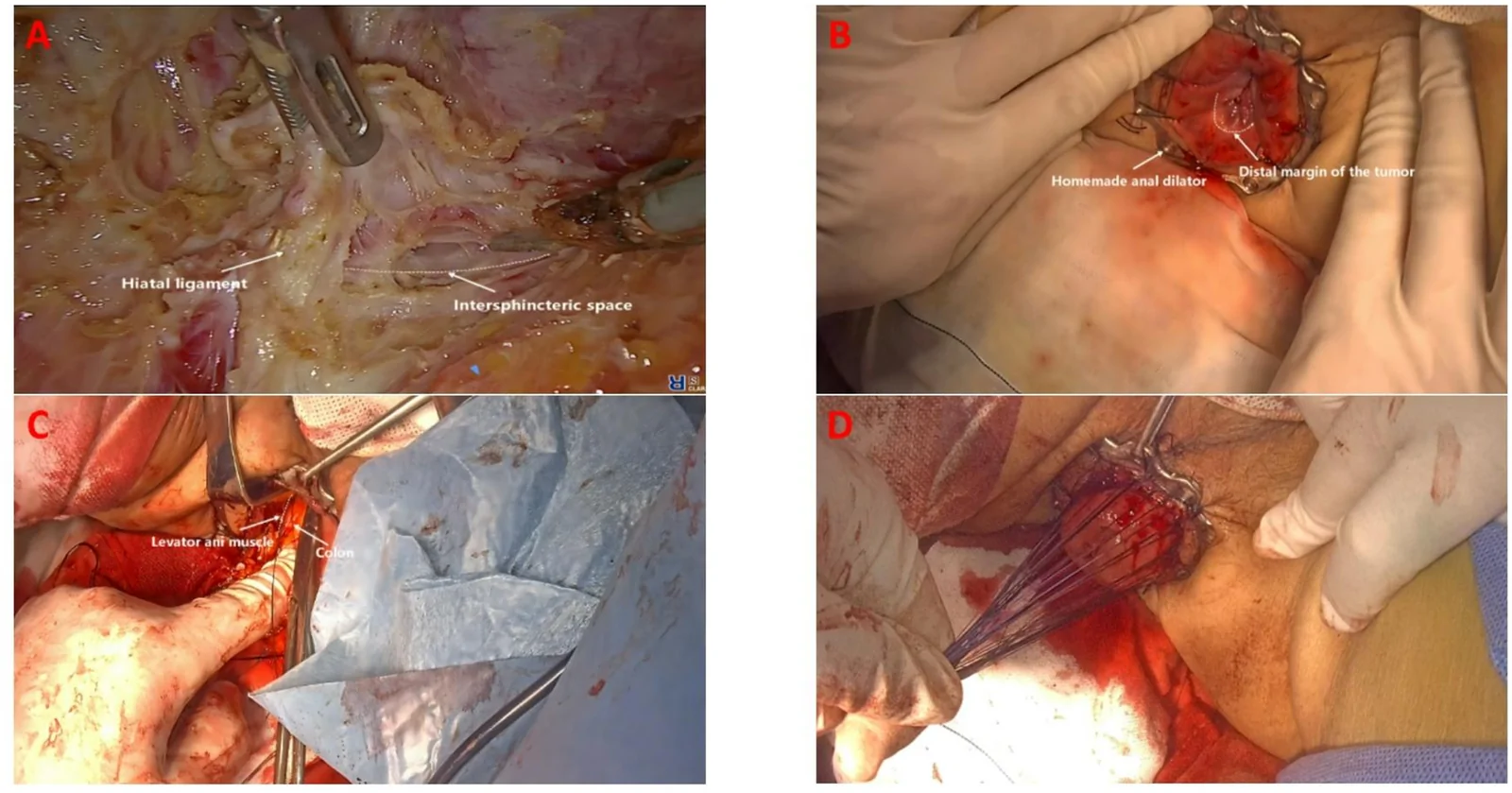
Modified Albert–Lembert coloanal anastomosis. **A **Mobilization of the distal rectum along the intersphincteric space. **B **The anus is retracted and secured using a custom-made anal retractor. **C **Following transection of the proximal bowel, the seromuscular layer of the intestine, 5 cm proximal to the resection margin, is secured to the levator hiatus. **D **Vertical mattress sutures are placed in four quadrants at the level of the intersphincteric groove to approximate the colonic stump and the external anal sphincter. The anastomosis is then reinforced with interrupted full-thickness sutures

Under general anesthesia, patients were placed in the lithotomy position. A 3D ultra-high-definition laparoscopic surgical system (Karl Storz, Tuttlingen, Germany) was used for all procedures. After abdominal exploration, laparoscopic total mesorectal excision was performed using a medial-to-lateral approach. Lymph node station 253 was dissected while the left colic artery (LCA) was preserved. The roots of the superior rectal artery (SRA) and sigmoid arteries were ligated. The sigmoid colon and mesorectum were then anatomically mobilized, and the splenic flexure was mobilized cephalad to the level of the inferior border of the pancreas. Using the “flip-over technique” of the STORZ laparoscopic surgical platform, the hiatal ligament was visualized and transected to access the intersphincteric space (Fig. 2A). The mesorectum was trimmed and mobilized, and lymph node stations 242 and 252 were dissected. The left colon was fully mobilized to the splenic flexure, and the proximal rectum and colon were positioned anatomically without twisting. In accordance with the International Guideline on Natural Orifice Specimen Extraction Surgery (NOSES) for colorectal cancer (2023 version) [13], povidone-iodine- or saline-soaked gauze was placed at the pelvic floor to prevent intra-abdominal contamination during the transanal phase.

##### Transanal procedure

The perineum was disinfected, and the intestinal lumen was thoroughly irrigated with a diluted povidone-iodine solution. After adequate anal dilation, a custom-made anal retractor was secured with eight interrupted sutures at the level of the intersphincteric groove to ensure full exposure (Fig. 2B). Approximately 1 cm distal to the tumor, traction was established in four quadrants of the rectal mucosa and submucosa using 4 − 0 silk sutures. Transanal dissection was then performed cephalad through the intersphincteric space until it merged with the abdominal dissection plane. Once mobilized sufficiently, the colon was exteriorized through the anus, and the sigmoid colon was transected approximately 10 cm proximal to the tumor, followed by re-disinfection of the intestinal lumen and meticulous hemostasis of the resection margin.

##### Digestive tract reconstruction

**Fig. 3:**
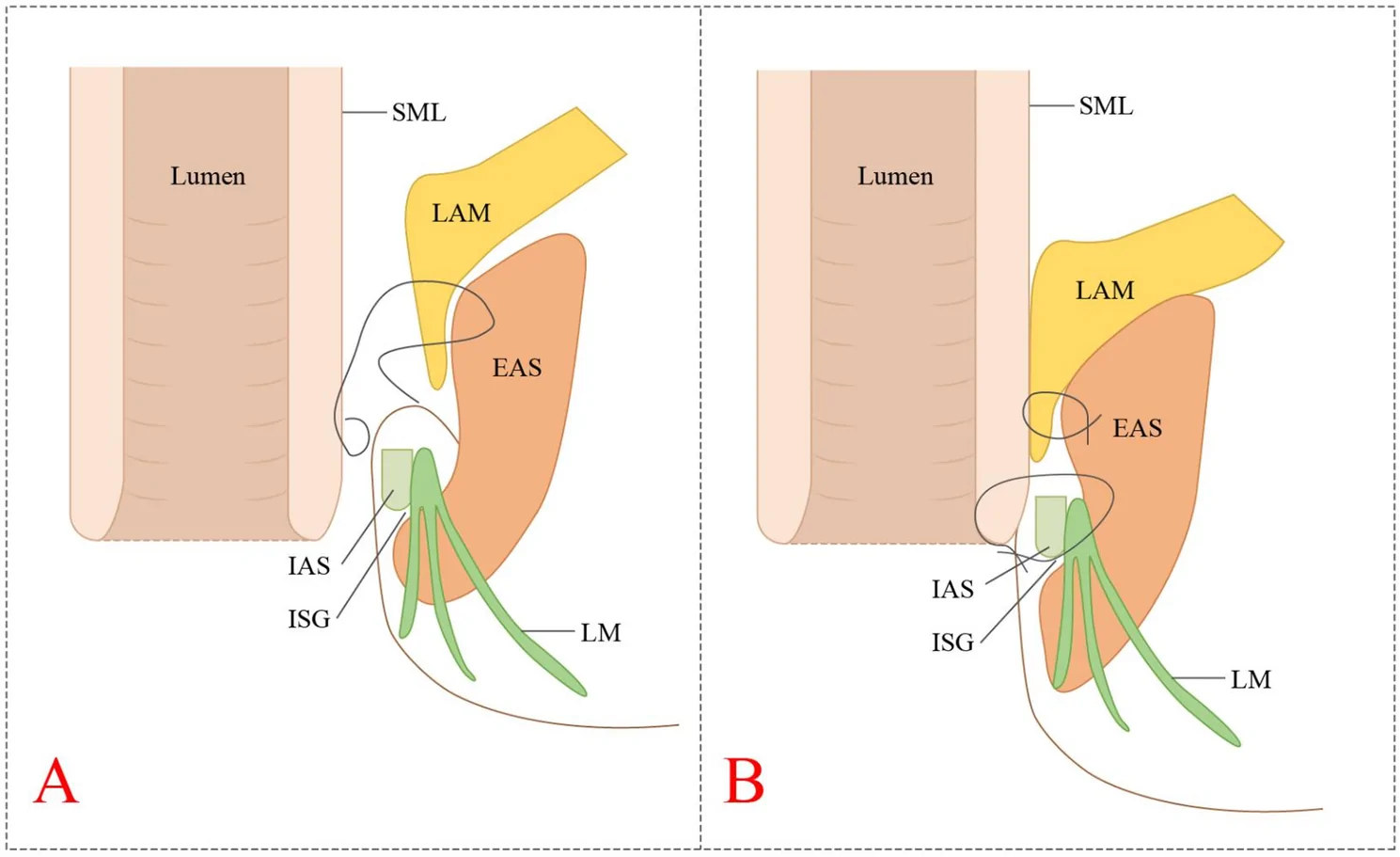
Schematic illustration of MALA. **A **The seromuscular layer of the proximal colon, approximately 5 cm from the resection margin, is anchored to the levator ani muscle to provide proximal fixation and reduce anastomotic tension. **B **The colonic stump is approximated to the external anal sphincter using vertical mattress sutures placed in four quadrants at the level of the intersphincteric groove, followed by reinforcement of the hand-sewn coloanal anastomosis. EAS, external anal sphincter; IAS, internal anal sphincter; ISG, intersphincteric groove; LAM, levator ani muscle; LM, longitudinal muscle; SML, seromuscular layer

Using 4 − 0 silk sutures on a small round-bodied needle, the seromuscular layer of the intestine, 5 cm proximal to the resection margin, was secured to the levator hiatus with interrupted sutures in four quadrants (Figs. 2C and 3A). Subsequently, the colonic stump was approximated to the external anal sphincter using vertical mattress sutures in four quadrants at the level of the intersphincteric groove (Figs. 2D and 3B). Finally, the anastomosis was reinforced with interrupted full-thickness 4 − 0 absorbable sutures, thus completing the hand-sewn end-to-end coloanal anastomosis. Laparoscopic peritoneal irrigation was performed, and a pelvic drain was placed before closure.

#### DCAA group

The patients were placed in the lithotomy position under general anesthesia. After establishment of pneumoperitoneum, laparoscopicexploration was performed to exclude distant metastasis. Laparoscopic total mesorectal excision was then performed according tooncological principles. The inferior mesenteric vessels and lymphovascular tissue were dissected, with preservation of the left colic arterywhenever feasible. The sigmoid colon and descending colon were mobilized sufficiently to ensure adequate bowel length for tension-freepull-through reconstruction. Care was taken to identify and protect the left ureter and autonomic pelvic nerves.

After completion of pelvic dissection to the level of the levator ani, the anal canal was exposed using an anal retractor. The distal rectumwas transected transanally under direct vision approximately 1 cm distal to the lower edge of the tumor. The specimen was extractedtransanally. The proximal bowel was then divided using a linear stapler at an adequate distance from the tumor, and the proximal colon waspulled through the anal canal without tension or twisting. Approximately 5 cm of the proximal colon was exteriorized beyond the analverge. The exteriorized colonic segment was fixed to the anorectal ring or perianal skin with interrupted sutures to prevent retraction. Theproximal staple line was opened to ensure decompression and luminal patency, and the exteriorized colon was covered with paraffin gauzeto prevent desiccation. Adequate perfusion and orientation of the exteriorized colon were confirmed at the end of the procedure. A pelvicdrain was placed, and the abdominal cavity was irrigated.

The planned second-stage perineal procedure was performed 1 to 2 weeks after the first surgery. During the second stage, the redundantexteriorized colonic segment was resected at the level of the anal verge, and a delayed hand-sewn coloanal anastomosis was completedusing interrupted absorbable sutures.

### Follow-up protocol

Patients were followed up in the outpatient clinic 30 days after discharge. Follow-up assessments included clinical examination, including digital rectal examination, complete blood count, liver and renal function tests, serum tumor marker measurements, chest radiography, and liver ultrasonography. Additional investigations, including computed tomography, magnetic resonance imaging, colonoscopy, or positron emission tomography–computed tomography, were performed when clinically indicated.

### Outcomes

The primary outcome was overall postoperative morbidity within 30 days after the first surgery. For patients in the DCAA group, adverse events occurring during the interval before delayed anastomosis or after the second-stage procedure within 30 days after the first surgery were included in the 30-day morbidity assessment. Overall postoperative morbidity was defined as any deviation from the expected postoperative course within 30 days after the first surgery that required medical, endoscopic, radiological, or surgical management.

Postoperative complications were graded by two colorectal surgeons according to the Clavien–Dindo classification [14]. Disagreements were resolved through discussion with a senior colorectal surgeon and the assessors were not blinded to the surgical procedure. When more than one complication occurred in the same patient, the most severe complication grade was used for classification of overall morbidity.

The definition of anastomotic leakage (AL) in this study was a defect in the intestinal wall integrity at the coloanal anastomotic site, which led to communication between the intraluminal and extraluminal compartments. A pelvic abscess without demonstrable anastomotic disruption or communication was recorded separately and was not classified as anastomotic leakage [15].

The secondary outcomes included operative time, intraoperative blood loss, R0 resection rate, postoperative hospital stay, 30-day mortality, Clavien–Dindo grade I–II complications, and Clavien–Dindo grade III or higher complications. Postoperative hospital stay was calculated from the date of the first surgery to the date of discharge; in the DCAA group, this interval included the planned second-stage perineal procedure when it was performed during the same hospitalization.

### Statistical analysis

Data analysis was performed using SPSS version 26.0. Continuous variables conforming to a normal distribution were expressed as mean ± standard deviation and compared using the independent-samples t-test. Non-normally distributed continuous variables were presented as median with range and compared using the Mann–Whitney U test. Categorical variables were expressed as frequencies and percentages and compared using the chi-square test or Fisher’s exact test, as appropriate. All tests were two-sided, and a P value < 0.05 was considered statistically significant.

## Results

### Baseline characteristics

A total of 100 patients were included in this study, including 58 patients in the DCAA group and 42 patients in the MALA group. The baseline demographic and clinical characteristics are summarized in Table 1. No statistically significant differences were observed between the DCAA and MALA groups in age [61 years (range, 42–70) vs. 62 years (range, 45–70), *P* = 0.638], sex distribution (*P* = 0.738), BMI [24.1 kg/m² (range, 18.8–31.6) vs. 23.9 kg/m² (range, 19.2–31.2), *P* = 0.814], ASA physical status (*P* = 0.902), tumor T stage (*P* = 0.849), tumor size [3.0 cm (range, 2.0–5.0) vs. 3.5 cm (range, 1.5–5.0), *P* = 0.512], or tumor height from the anal verge [3.5 cm (range, 2.0–5.0) vs. 3.5 cm (range, 2.0–5.0), *P* = 0.935].

**Table 1:** Baseline demographic and clinical characteristics

Characteristics	DCAA (*n* = 58)	MALA (*n* = 42)	*P* value
Age, years, median (range)	61 (42–70)	62 (45–70)	0.638
Sex, n (%)			0.738
Male	34 (58.6)	26 (61.9)	
Female	24 (41.4)	16 (38.1)	
BMI, kg/m², median (range)	24.1 (18.8–31.6)	23.9 (19.2–31.2)	0.814
ASA score, n (%)			0.902
I	10 (17.2)	8 (19.0)	
II	40 (69.0)	28 (66.7)	
III	8 (13.8)	6 (14.3)	
Tumor T-stage, n (%)			0.849
T1	19 (32.8)	13 (31.0)	
T2	39 (67.2)	29 (69.0)	
Tumor size, cm, median (range)	3.0 (2.0–5.0)	3.5 (1.5–5.0)	0.512
Tumor height, cm, median (range)	3.5 (2.0–5.0)	3.5 (2.0–5.0)	0.935

Data are presented as median (range) or n (%), as appropriate. *BMI *body mass index, *ASA *American Society of Anesthesiologists, *DCAA *Turnbull–Cutait pull-through delayed coloanal anastomosis, *MALA *modified Albert–Lembert coloanal anastomosis

### Primary and secondary outcomes

The primary and secondary outcomes are shown in Table 2. The 30-day overall postoperative morbidity rates were 32.8% (19/58) in the DCAA group and 23.8% (10/42) in the MALA group, with no statistically significant between-group difference (*P* = 0.378). No statistically significant differences were observed between the DCAA and MALA groups in operative time (201.8 ± 28.6 vs. 206.5 ± 32.4 min, *P* = 0.463), intraoperative blood loss [60 mL (range, 30–150) vs. 60 mL (range, 30–110), *P* = 0.408], or the number of harvested lymph nodes [19 (range, 15–23) vs. 22 (range, 16–24), *P* = 0.356]. The distal and circumferential resection margins were negative in all patients, and R0 resection was achieved in both groups. No statistically significant difference was observed in pathological stage distribution between the DCAA and MALA groups (*P* = 0.844). The median postoperative hospital stay was 15 days (range, 13–21) in the DCAA group and 14 days (range, 12–20) in the MALA group, with no statistically significant difference (*P* = 0.225). No 30-day mortality occurred in either group (Table 2).

**Table 2:** Primary and secondary outcomes

Outcomes	DCAA group (*n* = 58)	MALA group (*n* = 42)	*P* value
Overall postoperative morbidity, n (%)	19 (32.8)	10 (23.8)	0.378
Operative time, min, mean ± SD	201.8 ± 28.6	206.5 ± 32.4	0.463
Intraoperative blood loss, mL, median (range)	60 (30–150)	60 (30–110)	0.408
Harvested lymph nodes, median (range)	19 (15–23)	22 (16–24)	0.356
Positive DRM, n (%)	0	0	–
Positive CRM, n (%)	0	0	–
R0 resection, n (%)	58 (100.0)	42 (100.0)	1.000
Pathological stage, n (%)			0.844
I	34 (58.6)	27 (64.3)	
II	10 (17.2)	6 (14.3)	
III	14 (24.1)	9 (21.4)	
Postoperative hospital stay, days, median (range)	15 (13–21)	14 (12–20)	0.225
30-day mortality, n (%)	0	0	–

Data are presented as mean ± standard deviation, median (range), or n (%), as appropriate. *DCAA *Turnbull–Cutait pull-through delayed coloanal anastomosis, *MALA *modified Albert–Lembert coloanal anastomosis, *DRM *distal resection margin, *CRM *circumferential resection margin

### Details of postoperative complications

Details of postoperative complications within 30 days after the first surgery according to the Clavien–Dindo classification are presented in Table 3. No statistically significant differences were observed between the DCAA and MALA groups in Clavien–Dindo grade I–II morbidity (19.0% [11/58] vs. 16.7% [7/42], *P* = 0.799) or grade III or higher morbidity (13.8% [8/58] vs. 7.1% [3/42], *P* = 0.350). In the DCAA group, two patients developed major anastomotic leakage and one developed a pelvic abscess; all three underwent transabdominal lavage and negative-pressure drainage with antibiotic therapy. One patient underwent endoscopic hemostasis for anastomotic bleeding, and one required unplanned reoperation for retraction of the exteriorized colonic segment. Exteriorized colonic ischemia or necrosis occurred in three patients; one case of localized necrosis was managed conservatively, whereas two patients required temporary diverting ileostomy. In the MALA group, major complications included one case of anastomotic bleeding treated by endoscopic hemostasis, one pelvic abscess treated by transabdominal lavage, negative-pressure drainage, and antibiotics, and one anastomotic leakage requiring unplanned reoperation. Early ileostomy-related complications included one case each of stoma outlet obstruction, peristomal dermatitis, and high-output stoma, all of which were managed conservatively.

**Table 3:** Postoperative complications by Clavien–Dindo grade

Complications	DCAA group (*n* = 58)	MALA group (*n* = 42)	*P* value
Clavien–Dindo I–II, n (%)	11 (19.0)	7 (16.7)	0.799
Anastomotic leakage	4	2	
Anastomotic bleeding	1	1	
Anastomotic stricture	2	1	
Pelvic abscess	1	0	
Outlet obstruction	NA	1	
Peristomal dermatitis	NA	1	
High-output stoma	NA	1	
Urinary infection	2	0	
Exteriorized colon retraction	1	NA	
Clavien–Dindo III or higher, n (%)	8 (13.8)	3 (7.1)	0.350
Anastomotic leakage	2	1	
Anastomotic bleeding	1	1	
Pelvic abscess	1	1	
Exteriorized colon retraction	1	NA	
Exteriorized colon ischemia/necrosis	3	NA	

Data are presented as n. *DCAA *Turnbull–Cutait pull-through delayed coloanal anastomosis, *MALA *modified Albert–Lembert coloanal anastomosis, *NA *not applicable

## Discussion

In this study, we evaluated the short-term outcomes of MALA with diverting ileostomy and DCAA after laparoscopic ISR for low rectal cancer. The 30-day overall postoperative morbidity rates were 32.8% in the DCAA group and 23.8% in the MALA group, with no statistically significant between-group difference (*P* = 0.378). Similarly, no statistically significant differences were observed in Clavien–Dindo grade I–II morbidity (19.0% vs. 16.7%, *P* = 0.799) or grade III or higher morbidity (13.8% vs. 7.1%, *P* = 0.350). R0 resection was achieved in all patients, and no 30-day mortality occurred in either group. No statistically significant between-group differences were observed in operative time (201.8 ± 28.6 min vs. 206.5 ± 32.4 min, *P* = 0.463), intraoperative blood loss [60 mL (range, 30–150) vs. 60 mL (range, 30–110), *P* = 0.408], harvested lymph nodes [19 (range, 15–23) vs. 22 (range, 16–24), *P* = 0.356], or postoperative hospital stay [15 days (range, 13–21) vs. 14 days (range, 12–20), *P* = 0.225].

DCAA requires exteriorization of the proximal colon and a planned second-stage perineal procedure. During the interval between the first surgery and delayed anastomosis, the exteriorized colon requires careful management and may be complicated by ischemia, retraction, necrosis, patient discomfort, and nursing difficulty [16]. In the present study, Clavien–Dindo grade III or higher complications occurred in eight patients in the DCAA group, three of whom developed ischemia or necrosis of the exteriorized colonic segment. One patient had localized necrosis that was managed conservatively, whereas the other two required temporary diverting ileostomy. In these two patients, repeat DCAA was not attempted because of the unfavorable local tissue condition and marked edema. Further mobilization of the proximal colon and repeat pull-through exteriorization could have increased the risks of pelvic infection and anastomotic leakage. Therefore, based on the intraoperative assessment, temporary diverting ileostomy was considered the safer and more appropriate salvage strategy. Ischemia of the exteriorized colonic stump may be partly related to compression of the pulled-through colon and its mesentery by the anal sphincter complex, a mechanism referred to by some authors as the “guillotine effect”. To reduce the risks of stump ischemia, retraction, and patient discomfort during the interval between the two procedures, Bianco et al. developed the short stump and high anastomosis pull-through (SHiP) procedure. This technique involves extensive mobilization of the left colon, shortening of the exteriorized colonic stump, and completion of the delayed anastomosis at a higher level within the anal canal, with the aim of reducing compression of the colon and its mesentery. In their single-center retrospective study of 37 patients, no stump ischemia, retraction, or major anastomotic leakage was observed [17]. Bendib et al. subsequently reported a similar modified delayed coloanal anastomosis using a short exteriorized colonic stump and a high delayed anastomosis; among their 19 patients, only one developed stump ischemia [18]. Sage et al. reported colonic ischemia or necrosis in 9 of 85 patients (10.6%) undergoing laparoscopic DCAA without diverting ileostomy [19]. When complete necrosis of the exteriorized colon occurs, emergency surgery is required to resect the necrotic bowel and re-exteriorize a viable colonic segment; in severe cases, conversion to a permanent stoma may be necessary. Studies have reported that 4–20% of patients undergoing DCAA develop permanent stomas due to colonic ischemia [7, 20, 21].

Complications after one-stage coloanal anastomosis remain an important concern, particularly in patients undergoing ISR for rectal cancer. A previous meta-analysis reported that the 30-day postoperative morbidity rate after ISR was approximately 25.8%, including anastomotic leakage in 9.1%, pelvic sepsis in 2.4%, and anastomotic stricture in 2.7% of patients [22]. To improve anastomotic stability after ISR, we performed modified Albert–Lembert one-stage coloanal anastomosis (MALA) with diverting ileostomy. MALA reinforces a one-stage hand-sewn coloanal anastomosis through fixation of the proximal colonic seromuscular layer to the levator hiatus and approximation of the colonic stump to the external anal sphincter. This technique is designed to reduce anastomotic tension, prevent proximal colonic retraction, and stabilize the neorectal mucosa. The additional diverting ileostomy may protect the anastomosis during early healing and reduce the clinical consequences of anastomotic leakage.

In the present study, Clavien–Dindo grade III or higher complications occurred in three patients in the MALA group, including one case of anastomotic leakage requiring reoperation. Notably, this patient had an underlying autoimmune disease and had received long-term corticosteroid therapy, which may have adversely affected tissue healing and contributed to the development of anastomotic leakage. The overall incidence of anastomotic leakage in the MALA group was 7.1% (3/42), which appeared to be lower than the average rate reported in previous studies [22]. The relatively low rate of anastomotic leakage observed in the present study may be explained by several factors. First, the left colic artery was preserved whenever feasible in all patients, which may have helped maintain adequate blood supply to the distal colon and anastomotic site. Adequate perfusion is widely recognized as a key factor in reducing the risk of anastomotic leakage. Previous meta-analyses have suggested that low ligation of the inferior mesenteric artery with preservation of the left colic artery is associated with a lower risk of anastomotic leakage compared with high ligation, without clear evidence of compromised oncological outcomes [23, 24]. Second, none of the included patients received preoperative local radiotherapy. Previous studies have shown that neoadjuvant radiotherapy may increase the risk of postoperative anastomotic leakage in patients with rectal cancer [25, 26]. In addition, diverting ileostomy has been widely used to mitigate the risk and clinical consequences of anastomotic leakage after low rectal or coloanal anastomosis. A randomized multicenter trial by Matthiessen et al. demonstrated that a defunctioning stoma significantly reduced symptomatic anastomotic leakage and urgent abdominal reoperation after low anterior resection for rectal cancer. Consistently, Gu and Wu performed a meta-analysis of 13 studies involving 8,002 patients and found that defunctioning stoma significantly reduced both postoperative anastomotic leakage and leakage-related reoperation after low anterior resection with total mesorectal excision [27, 28].

In the present cohort, early stoma-related complications in the MALA group included one case of stoma outlet obstruction, one case of peristomal dermatitis, and one case of high-output stoma. All of these events were classified as Clavien–Dindo grade I–II complications and were managed conservatively. The patient with stoma outlet obstruction improved after early gastrointestinal decompression and subsequent dietary modification. Abe et al. evaluated 125 patients who underwent rectal resection with anastomosis and elective diverting ileostomy and reported stoma outlet obstruction in 20 patients (16.0%). In most cases, the complication developed approximately 9 days after surgery, and the obstructive symptoms resolved within 4 days after trans-stomal decompression [29]. High-output stoma represents another clinically relevant early complication, as excessive ileostomy output may result in dehydration, electrolyte disturbance, and renal dysfunction. In a systematic review and meta-analysis of 22 studies involving 19,485 patients undergoing colorectal cancer resection, Borucki et al. reported a pooled prevalence of dehydration of 9.0% following diverting ileostomy [30]. Accordingly, daily monitoring of stoma output, timely fluid and electrolyte replacement, dietary adjustment, and antidiarrheal medication are important during the early postoperative period. Peristomal dermatitis is another common complication of diverting ileostomy. In a retrospective study of 491 patients with colorectal cancer and defunctioning ileostomy, He et al. reported that 17.3% developed peristomal dermatitis within 1 month after ileostomy formation. The incidence was significantly lower among patients who participated in a postoperative stoma education program than among those who did not (11.1% vs. 20.4%, *P* = 0.011). These findings suggest that structured patient education, appropriate appliance fitting, prevention of effluent leakage, and specialized stoma care may help reduce the risk of early peristomal skin complications [31].

In the present study, the number of harvested lymph nodes was comparable between the DCAA and MALA groups [19 (range, 15–23) vs. 22 (range, 16–24), *P* = 0.356]. Distal and circumferential resection margins were negative in all patients, and R0 resection was achieved in both groups, indicating comparable oncological adequacy of resection. Nevertheless, postoperative oncological surveillance remains an important consideration. Both procedures involved transanal pull-through procedures. Routine colonoscopy or sigmoidoscopy allows direct visualization of the luminal surface of the reconstructed colon and the neo-coloanal anastomosis. However, tissue within the intersphincteric plane or perianastomotic region may not be fully accessible to routine endoscopic examination. Therefore, endoscopic surveillance alone may be insufficient for comprehensive assessment of the local operative field; when local recurrence is suspected, digital rectal examination combined with cross-sectional pelvic imaging, particularly magnetic resonance imaging, may provide important complementary information. Although the TURNBULL-BCN randomized trial demonstrated comparable 3-year oncological outcomes between DCAA and conventional hand-sewn coloanal anastomosis with diverting ileostomy [32], longer-term studies incorporating standardized multimodal surveillance strategies are still warranted.

This study has several limitations. First, its retrospective, nonrandomized design and the absence of robust multivariable or propensity-score adjustment may have introduced selection bias and residual confounding. Second, the marked imbalance in diversion status was the principal confounding factor. Third, the lack of an a priori sample-size calculation and the relatively small sample size limited the statistical power. Fourth, the inclusion of only patients with early T-stage tumors who had not received neoadjuvant therapy limits the generalizability of the findings to patients with prior neoadjuvant treatment or more complex pelvic anatomy. Fifth, the short follow-up period precluded comprehensive assessment of long-term oncological, functional, and ileostomy-closure-related outcomes. Finally, this was a single-center study performed by an experienced colorectal surgical team, which may limit its external validity. Future large-scale prospective multicenter studies should compare MALA with conventional one-stage hand-sewn coloanal anastomosis under the same diversion strategy and incorporate standardized functional and quality-of-life assessments, comprehensive evaluation of stoma-closure-related outcomes, and longer oncological follow-up.

## Conclusions

MALA with diverting ileostomy is a technically feasible sphincter-preserving reconstructive strategy in selected patients undergoing laparoscopic ISR for low rectal cancer. Compared with conventional DCAA, MALA avoids management of an exteriorized colonic segment and a planned second-stage perineal procedure. No statistically significant differences in 30-day overall postoperative morbidity or complication severity were observed between the two groups. Larger prospective multicenter studies with longer follow-up are warranted to evaluate functional, quality-of-life, stoma-closure-related, and long-term oncological outcomes.

## Data Availability

The datasets used and analyzed in this study are available from the corresponding author upon reasonable request.

## Abbreviations

AJCC: American Joint Committee on Cancer
ASA: American Society of Anesthesiologists physical status classification
BMI: Body mass index
CRM: Circumferential resection margin
DCAA: Turnbull–Cutait pull-through delayed coloanal anastomosis
DRM: Distal resection margin
EAS: External anal sphincter
IAS: Internal anal sphincter
ISG: Intersphincteric groove
ISR: Intersphincteric resection
LAM: Levator ani muscle
LM: Longitudinal muscle
MALA: Modified Albert–Lembert coloanal anastomosis
NA: Not applicable
SD: Standard deviation
SML: Seromuscular layer
TNM: Tumor–node–metastasis
